# Significance of CD80 as a Prognostic and Immunotherapeutic Biomarker in Lung Adenocarcinoma

**DOI:** 10.1007/s10528-023-10343-7

**Published:** 2023-03-09

**Authors:** Wei Feng, Ziyi He, Liang Shi, Zheng Zhu, Haitao Ma

**Affiliations:** First Affiliated Hospital of Soochow University, Suzhou, China

**Keywords:** Lung adenocarcinoma (LUAD), CD80, Prognosis, Immune response, Immune checkpoint inhibitors (ICIs), Small-molecule drugs

## Abstract

Lung adenocarcinoma (LUAD) is the primary cause of death among pulmonary cancer patients. Upregulation of CD80 may interact with cytotoxic T lymphocyte antigen 4 (CTLA4) to promote tumor progression and provide a potential target for biological antitumor therapy. However, the role of CD80 in LUAD is still unclear. To investigate the function of CD80 in LUAD, we collected transcriptomic data from 594 lung samples from The Cancer Genome Atlas of America (TCGA) database, along with the corresponding clinical information. We systematically explored the role of CD80 in LUAD using bioinformatics methods, including GO enrichment analysis, KEGG pathway analysis, Gene Set Enrichment Analysis (GSEA), co-expression analysis, and the CIBERSORT algorithm. Finally, we investigated the differences between the two subgroups of CD80 expression in terms of some drug sensitivity, using the pRRophetic package to screen small molecular drugs for therapeutic use. A predictive model based on CD80 for LUAD patients was successfully constructed. In addition, we discovered that the CD80-based prediction model was an independent prognostic factor. Co-expression analysis revealed 10 CD80-related genes, including oncogenes and immune-related genes. Functional analysis showed that the differentially expressed genes in patients with high CD80 expression were mainly located in immune-related signaling pathways. CD80 expression was also associated with immune cell infiltration and immune checkpoints. Highly expressing patients were more sensitive to several drugs, such as rapamycin, paclitaxel, crizotinib, and bortezomib. Finally, we found evidence that 15 different small molecular drugs may benefit the treatment of LUAD patients. This study found that elevated CD80 pairs could improve the prognosis of LUAD patients. CD80 is likely to be a potential as a prognostic and therapeutic target. The future use of small molecular drugs in combination with immune checkpoint blockade to enhance antitumor therapy and improve prognosis for LUAD patients is promising.

**Supplementary Information:**

The online version contains supplementary material available at 10.1007/s10528-023-10343-7.

## Introduction

Lung adenocarcinoma (LUAD) is the leading cause of death among pulmonary cancer patients, with over 1 million deaths worldwide each year (Chen et al. 2020). With the popularity of low-dose spiral computed tomography (CT) in lung cancer screening, more and more pulmonary cancer patients are diagnosed early and completely resected, thus improving prognosis. Meanwhile, biological antitumor therapy has achieved remarkable results. With the use of emerging molecular-targeted therapies and ICIs, as well as conventional chemotherapy and radiotherapy, the prognosis of advanced and metastatic LUAD has improved significantly (Yang et al. 2020a, b; Zhang et al. 2019). However, the efficacy of biological antitumor therapy for LUAD needs to be further improved due to drug resistance, tumor heterogeneity, and metastasis (Quintanal-Villalonga et al. 2020). To better improve the prognosis of LUAD, it is important to investigate critical biomarkers for immunotherapy and optimize combination therapy options.

CD80 is a cell surface receptor that is activated by binding to CD28 or cytotoxic T lymphocyte antigen 4 (CTLA4). Activated proteins further induce T-cell proliferation and cytokine production. A decade ago, Freeman and colleagues discovered that CD80 and PD-L1 are interrelated (Butte et al. 2007). CD80-PD-L1 interactions occur on the same cell membrane (Chaudhri et al. 2018). *Cis* interactions between CD80 and PD-L1 preclude binding of the latter to PD-1, thereby enhancing the immune response (Sugiura et al. 2019). The two recent small cell lung cancer-related trials (CASPIAN (Paz-Ares et al. 2019) and IMpower133 (Horn et al. 2018)) showed a survival benefit to adding anti-PD-L1 (αPD-L1) therapy to chemotherapy over chemotherapy alone. In non-small cell lung carcinoma (NSCLC), too, immunotherapy may prolong survival in NSCLC patients when used in combination with chemotherapy (Li et al. 2020). Soluble CD80 as a therapeutic agent, combined with αPD-1 monoclonal antibody therapy, may be more effective for cancer patients than αPD-1 monotherapy. Therefore, targeted manipulation of *cis*-PD-L1/CD80 interactions may provide a new strategy for biological antitumor therapy (Sugiura et al. 2019).

Previous studies have shown that elevated CD80 expression prevents PD-L1 from binding to PD-1, thereby enhancing the immune response. However, the clinical significance of CD80 in LUAD remains unclear. We systematically analyzed the RNA sequencing (RNAseq) data from 59 normal and 535 LUAD samples obtained from TCGA database, in combination with their clinical characteristics, to further make the role of CD80 in LUAD clear. We focused on the expression profile of CD80 in LUAD and its prognostic value by bioinformatics analysis. We then successfully constructed a nomogram based on CD80 expression that could broadly predict the prognosis of LUAD patients. Moreover, we determined the correlation between CD80 expression and the wider immune microenvironment, which provides a theoretical basis for novel ICI strategies. Finally, we screened 15 small-molecule drugs that may improve prognosis for LUAD patients.

## Methods

### Data Sourcing

With the purpose of exploring differential CD80 expression in non-tumor and tumor samples, we downloaded transcriptomic data from the TCGA (https://portal.gdc.cancer.gov/) database for 594 samples, including 59 normal samples and 535 LUAD tumor samples. Clinical information derived from TCGA of 594 LUAD specimens were included to study the correlation between clinical features and CD80 expression.

### TCGA Data Processing

Due to lack of survival status and/or clinical information, 13 of the 535 LUAD tumor samples were excluded. The significance of CD80 on the prognosis of LUAD was examined in the remaining 522 cases using Kaplan–Meier survival curves with log-rank tests. Next, we used univariate and multifactorial Cox proportional risk models to calculate hazard ratios (HRs) for CD80 expression levels and other clinical features in LUAD. Thus, we found that CD80 was an independent risk factor affecting the prognosis of LUAD patients and its elevated expression resulted in improved overall survival (OS).

### Clinical Correlation Analysis

We used the “Limma” package in R for clinical correlation analysis and a clinical correlation heat map was drawn by the “ComplexHeatmap” package. According to the median CD80 expression, we classified LUAD patients from TCGA into high and low expression groups. Using “Limma” package to discuss the differences between the two subgroups, we then explored the association between clinical characteristics and CD80 expression.

### Nomogram Construction Based on CD80 Expression and Clinical Features

To determine whether CD80 expression, as a single factor, could predict the prognosis of LUAD patients, we performed univariate and multifactorial Cox regression analyses in combination with other clinical variables. The prognosis-related nomogram survival risk assessment model was applied to clinical variables and CD80 expression to assess the likelihood of 1-, 3-, and 5-year survival in LUAD patients. Based on bootstrap resampling method (Zhou et al. 2019), we assessed the predictive function of the nomogram by the calibration curve.

### Functional Enrichment Analyses

Gene Ontology (GO) enrichment analysis for both subgroups, including molecular function (MF), biologic process (BP), and cellular components (CC), was performed through the ClusterProfiler package. Kyoto Encyclopedia of Genes and Genomes (KEGG) pathway analysis was conducted with the same method for both subgroups.

We performed GSEA analysis using the “Limma” package in R (version 4.1.2) to assess the correlation between target genomes and specific genomes. The median CD80 expression level was used as a cut-off value to classify the target genes into high and low expression. In addition, we performed co-expression analysis of the target genes. The correlation coefficients of the target genes with other important genes were calculated using R (version 4.1.2). We then plotted a circular graph to more visually demonstrate the possible interactions of CD80 with other genomes in LUAD.

### Immune Cell Infiltration Analysis

A growing number of studies have confirmed that immune tumor infiltration is involved in cancer onset, progression, metastasis, and immune escape and is associated with prognosis. We first used ESTIMATE algorithm to calculate the immune score, stromal score, and estimate score of each sample. And then, we accordingly used algorithms such as CIBERSORT to assess the difference in the level of immune cell infiltration between the high CD80 expression group and the low CD80 expression group (results of immune cell infiltration for each sample are shown in Supplementary file 1). In order to predict the therapeutic effect of ICIs, we also explored the relationship between CD80 expression and the expression of several immune checkpoints, such as CTLA4 and CD274 (PD-L1). Next, we performed a differential analysis of TMB between the two subgroups and analyzed the relationship between CD80 and TMB. Finally, the sensitivity of LUAD patients to immune checkpoint inhibitors was predicted by the TCIA database (https://tcia.at/).

### Drug Sensitivity by pRRophetic

We used the “pRRophetic” package in R to study the drug sensitivity differences between the two subgroups. We predicted the sensitivity of the drug accurately by analyzing the half-maximal inhibitory concentration (IC50).

### Statistical Analysis

We used the “survival” package for Cox regression analysis to build survival models. The “LIMMA” package was used for normalization and analysis of variance. The “ESTIMATE” package was used to calculate tumor scores. The Wilcoxon rank-sum test was used for comparison between two groups, and the Kruskal–Wallis test was used for comparison between two or more groups. Statistical analysis was performed using R software (version 4.1.2). *P* values < 0.05 were considered statistically significant.

## Results

### Contributions of CD80 Mutation in the Prognosis of LUAD Patients Analyzed in TCGA

**Fig. 1:**
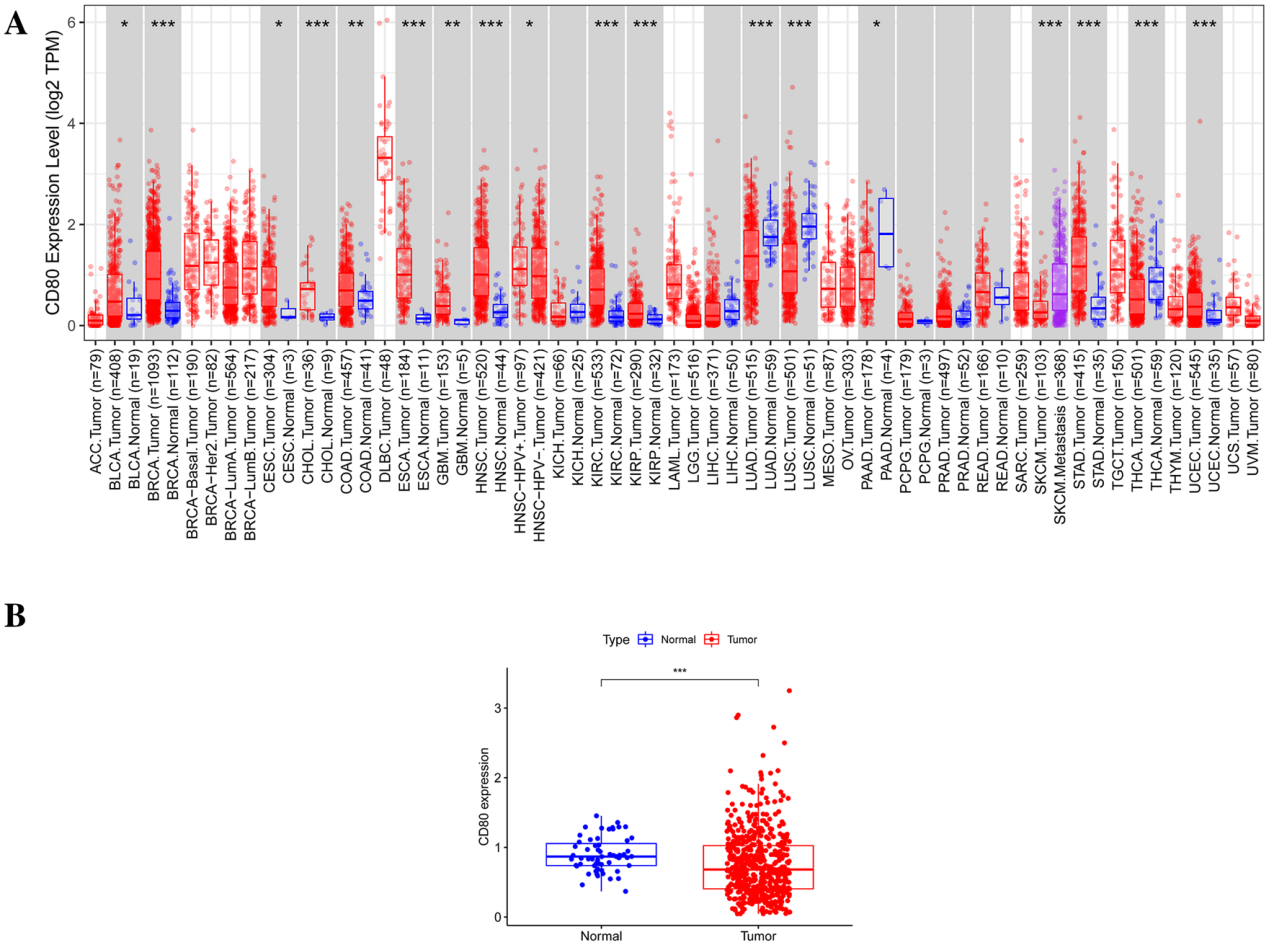
Expression of CD80 in normal and tumor tissues. **A** CD80 expression in normal tissues and cancer; **B** CD80 expression in normal lung tissues (*N* = 59) and LUAD (*N* = 535). *p < 0.05; **p < 0.01; and ***p < 0.0001

In the TIMER database (http://timer.cistrome.org/), we obtained information on the differential expression of CD80 in pan-cancerous tumor tissues. CD80 was highly expressed in many malignant tumors, whereas in several others, including in LUAD, it was expressed at lower levels than in normal tissues (Fig. 1A). The differences between these were significant (*p* < 0.05). LUAD was of particular interest to us in this study. Therefore, with the TCGA database, we separately calculated the differential expression of CD80 in LUAD tumor tissues and paracancerous tissues. As the results suggested, the expression of CD80 was lower in LUAD tumor tissues than in normal tissues (*p* < 0.05) (Fig. 1B).

**Fig. 2:**
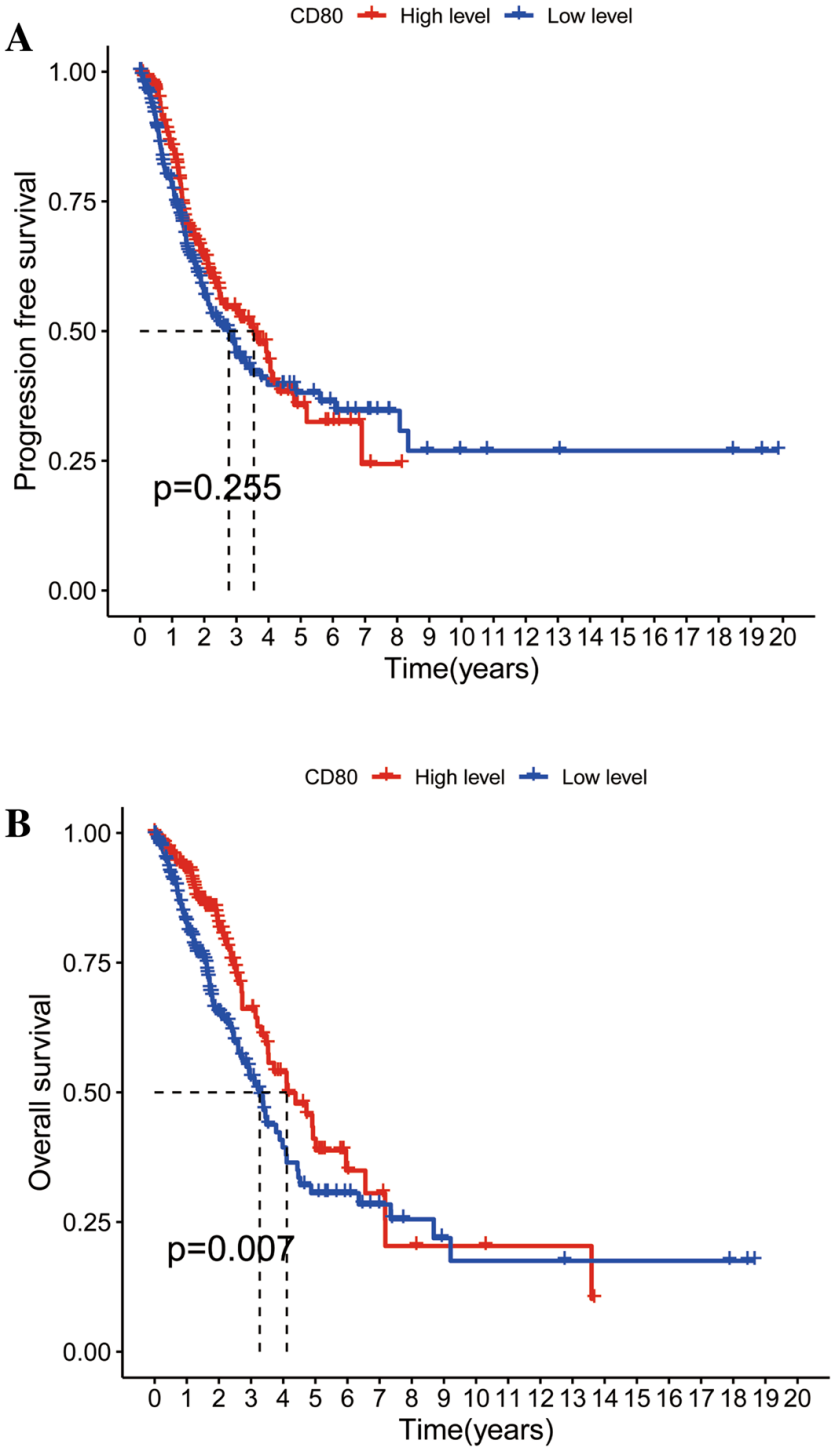
Survival analysis of LUAD patients in the CD80 high expression group and CD80 low expression group. **A** Progression-free survival analysis of LUAD patients in CD80 high expression group and CD80 low expression group, *p* = 0.255; **B** overall survival analysis of LUAD patients in CD80 high expression group and CD80 low expression group, *p* = 0.007

Moreover, survival data of TCGA-LUAD were downloaded from the TCGA database. Based on median values of CD80 expression, we divided the samples from TCGA (594 cases) into two subgroups, the high expression group and the low expression group. Kaplan–Meier analysis showed that the overall survival (OS) period of patients in the high CD80 expression group (297 cases) was significantly longer than that of patients in the low CD80 expression group (297 cases; *p* = 0.007) (Fig. 2A). However, the progression-free survival (PFS) in the CD80 high expression group was not significantly different from that of patients in the CD80 low expression group (Fig. 2B, p = 0.255).

### Association Between CD80 Expression and Clinical Characteristics

**Fig. 3:**
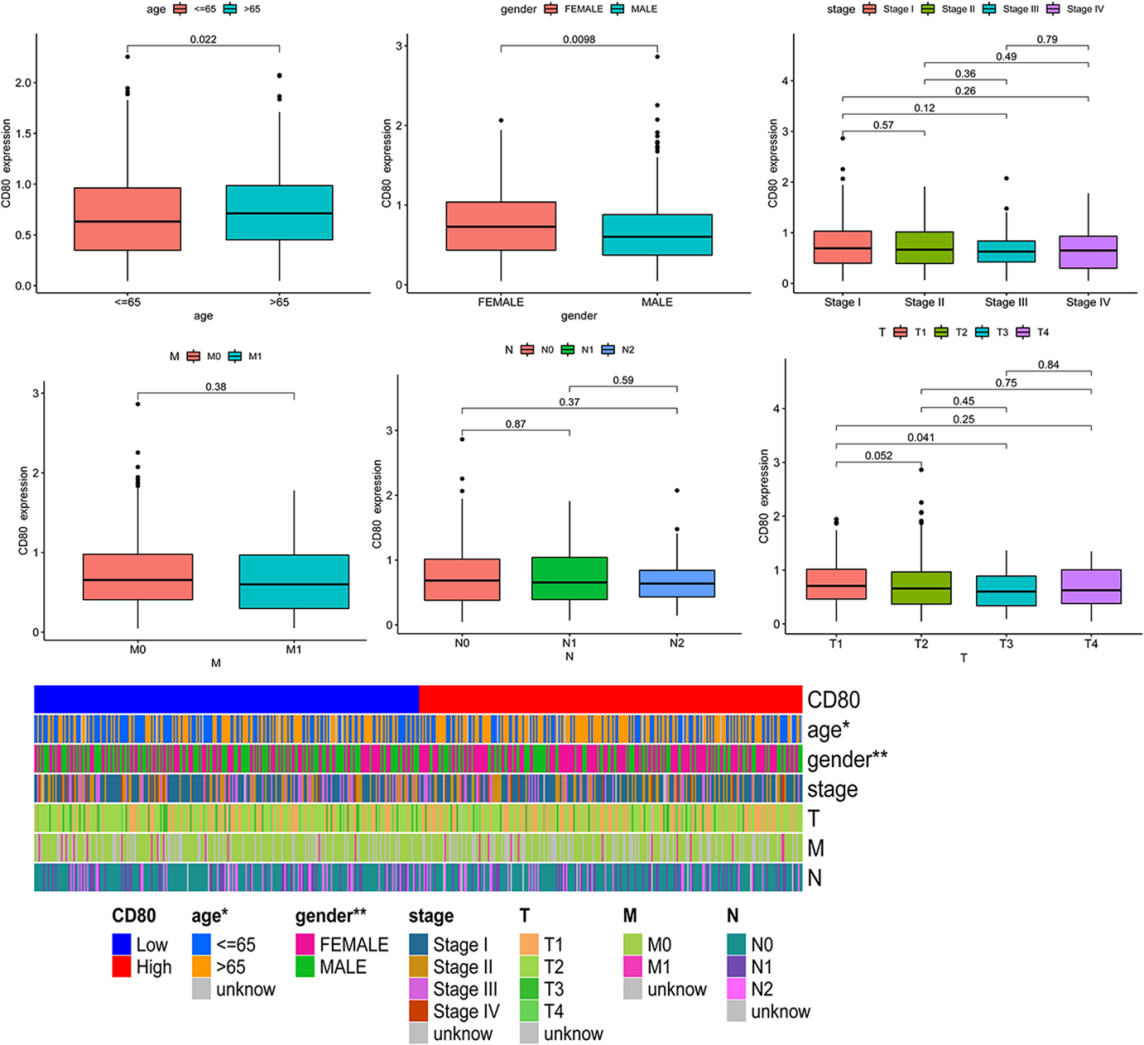
Correlation analysis of CD80 expression and clinical characteristics

On this basis, we further investigated the relation between CD80 expression and clinical traits, such as age, gender, stage, tumor (T), node (N), and metastasis (M). We found that CD80 was highly expressed in patients aged > 65 years (*p* = 0.022) and in female patients (*p* = 0.0098), while we did not find any significant differences in CD80 expression in terms of stage, tumor, node, or metastasis (Fig. 3).

### Independent Prognostic Analysis

**Fig. 4:**
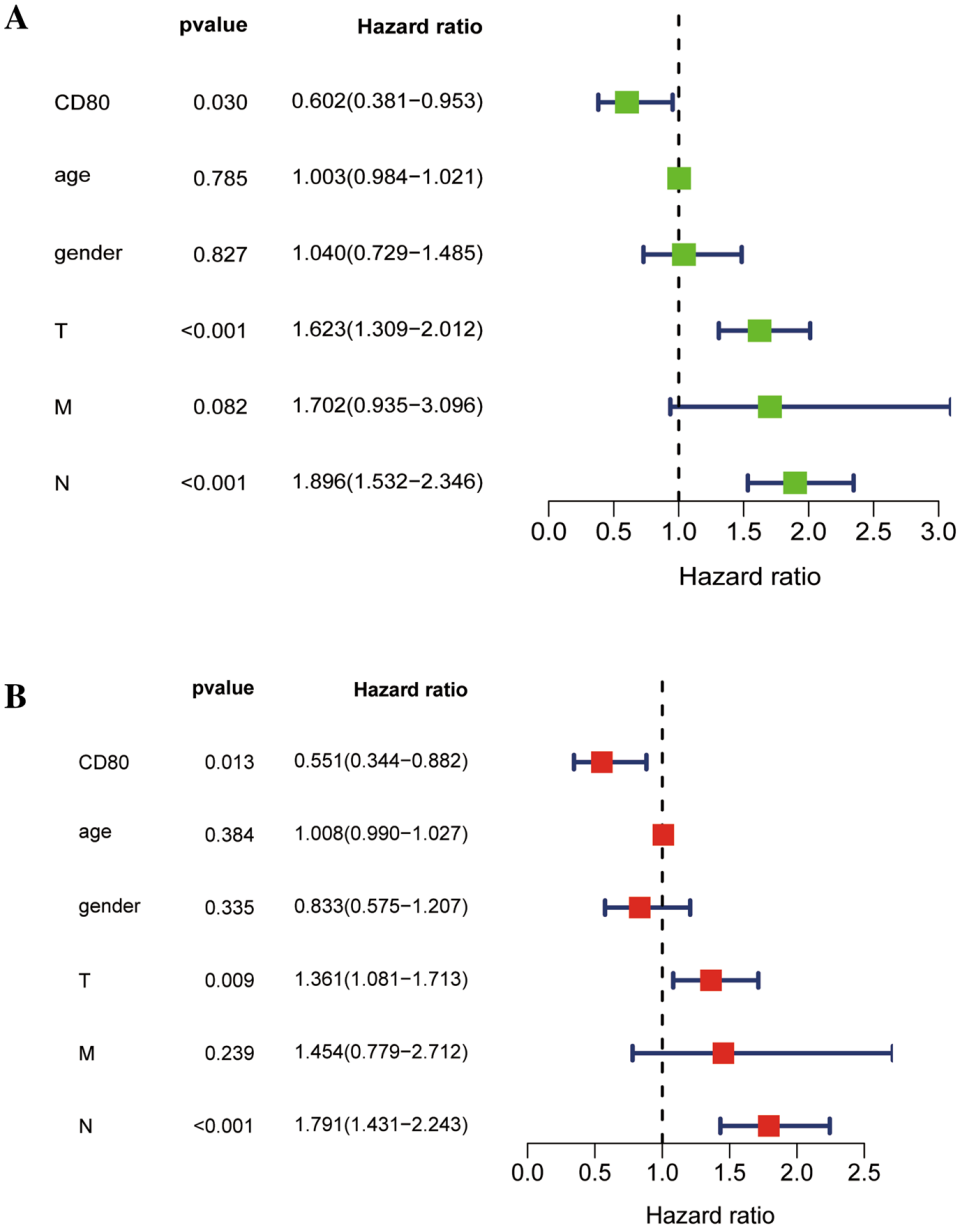
CD80 was an independent prognostic factor for LUAD in the TCGA set. **A** Correlations between CD80 for OS and clinicopathological factors by univariate Cox regression analysis; **B** correlations between CD80 for OS and clinicopathological factors by multivariate Cox regression analysis

To determine whether CD80 could be used as an independent prognostic indicator for LUAD, we performed univariate and multivariate COX analyses. Univariate analysis showed that CD80 expression, T stage, and N stage were significantly associated with prognosis in LUAD patients (Fig. 4A, *p* < 0.05). Multivariate analysis validated that CD80 expression, T stage, and N stage remained highly correlated with prognosis (Fig. 4B, *p* < 0.05). And as the results of the analysis showed, CD80 could be used as an independent prognostic biomarker for LUAD patients.

### Nomogram Construction

**Fig. 5:**
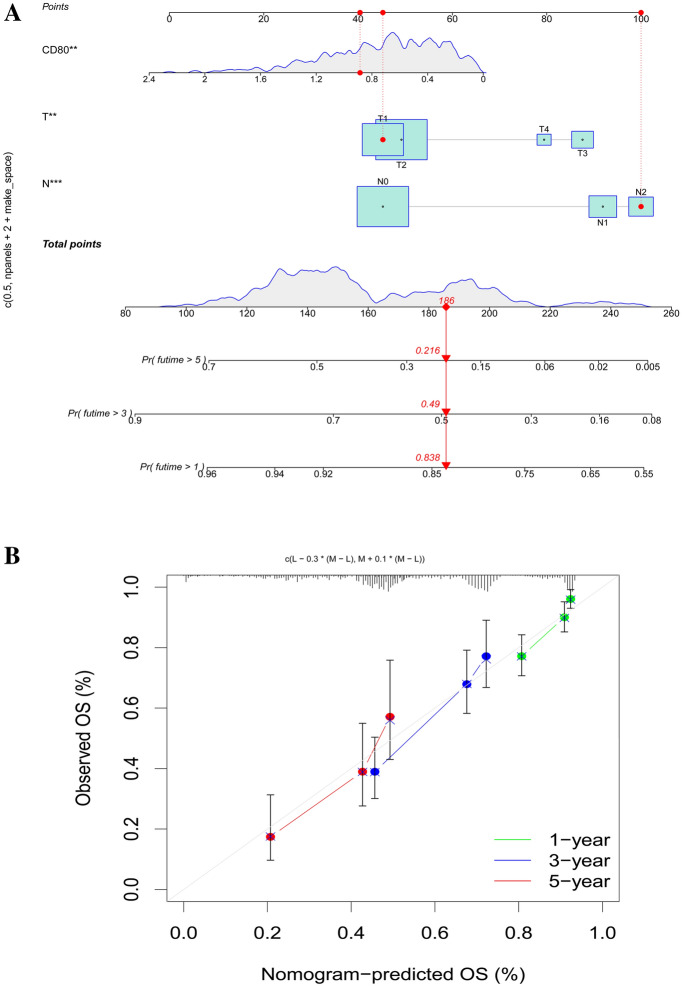
Construction of a nomogram. **A** Nomogram for predicting 1-, 3- or 5-year OS; **B** calibration plots for predicting 1-, 3-, or 5-year OS

According to the results of multivariate COX analyses, we constructed a nomogram consisting of CD80 expression and clinical characteristics to predict the survival of LUAD patients. Nomography predicted the 1-, 3-, and 5-year survival rate of LUAD patients (Fig. 5A). The calibration curve showed that the predicted values were in approximate correspondence with the actual patient survival rate (Fig. 5B).

### Co-expression Analysis of CD80

**Fig. 6:**
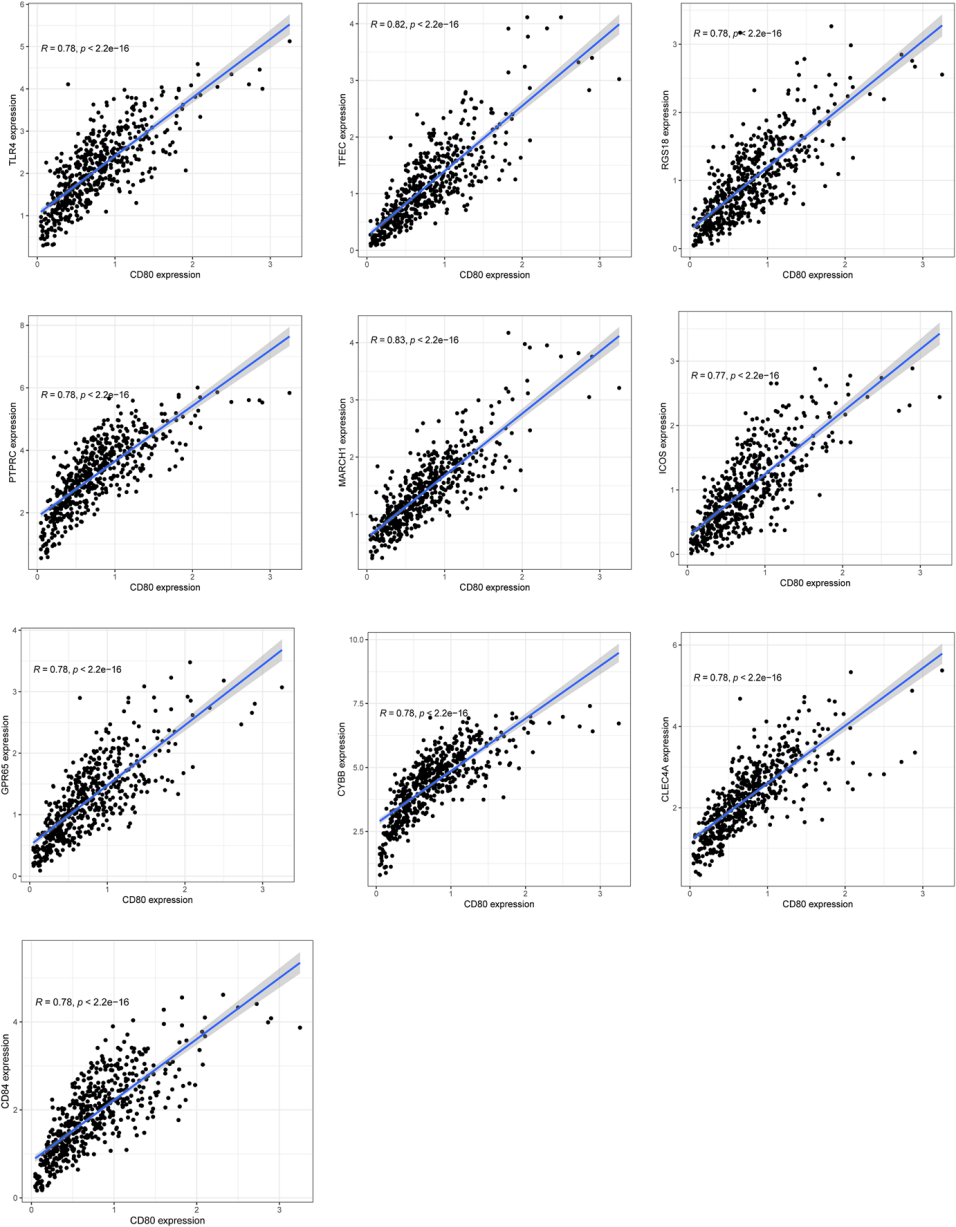
Co-expression curves of the top 10 genes positively associated with CD80 gene,* p* < 0.01

**Fig. 7:**
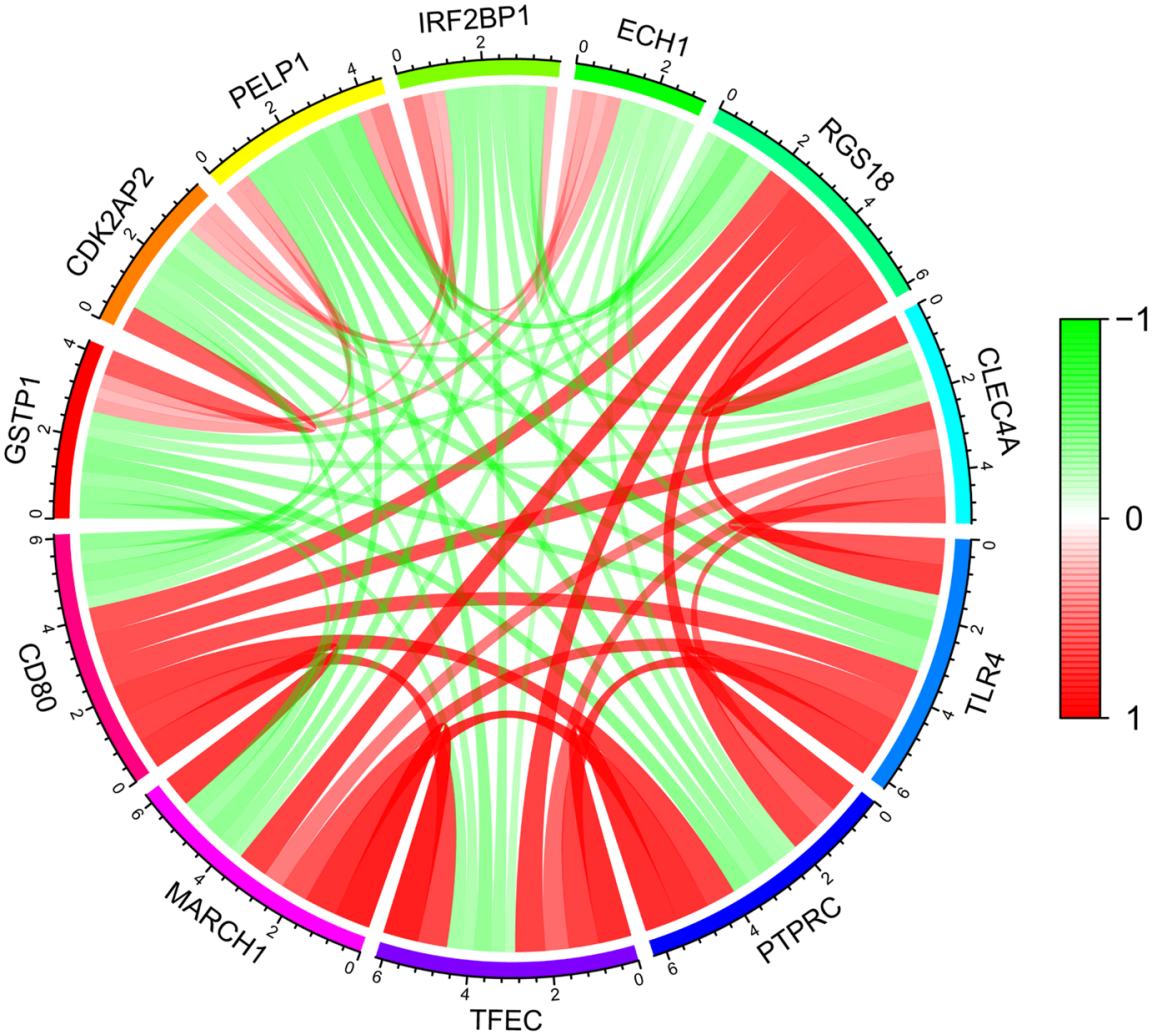
Circular plot of the top five genes positively and negatively correlated with the *CD80* gene. Green represents negative association, and red represents positive association (Color figure online)

To explore relationships between CD80 and other genes in TCGA database, we performed co-expression analysis and found a strong correlation between CD80 and 13,844 genes, with a correlation coefficient between − 0.6 and 0.8. Among them, *MARCH1*, *TFEC*, *PTPRC*, *TLR4*, *CLEC4A*, *RGS18*, *CD84*, *CYBB*, *GPR65*, and *ICOS* were positively correlated with CD80 and had the largest co-expression coefficients (Table 2, Fig. 6). We then mapped the co-expression circles of the 11 co-expressed genes most associated with CD80 (Fig. 7). As shown, *GSTP1*, *CDK2AP2*, *PELP1*, *IRF2BP1*, and *ECH1* were negatively correlated with CD80, while *MARCH1*, *TFEC*, *PTPRC*, *TLR4*, *CLEC4A*, and *RGS18* were positively correlated with CD80.

### Functional Enrichment Analysis

**Fig. 8:**
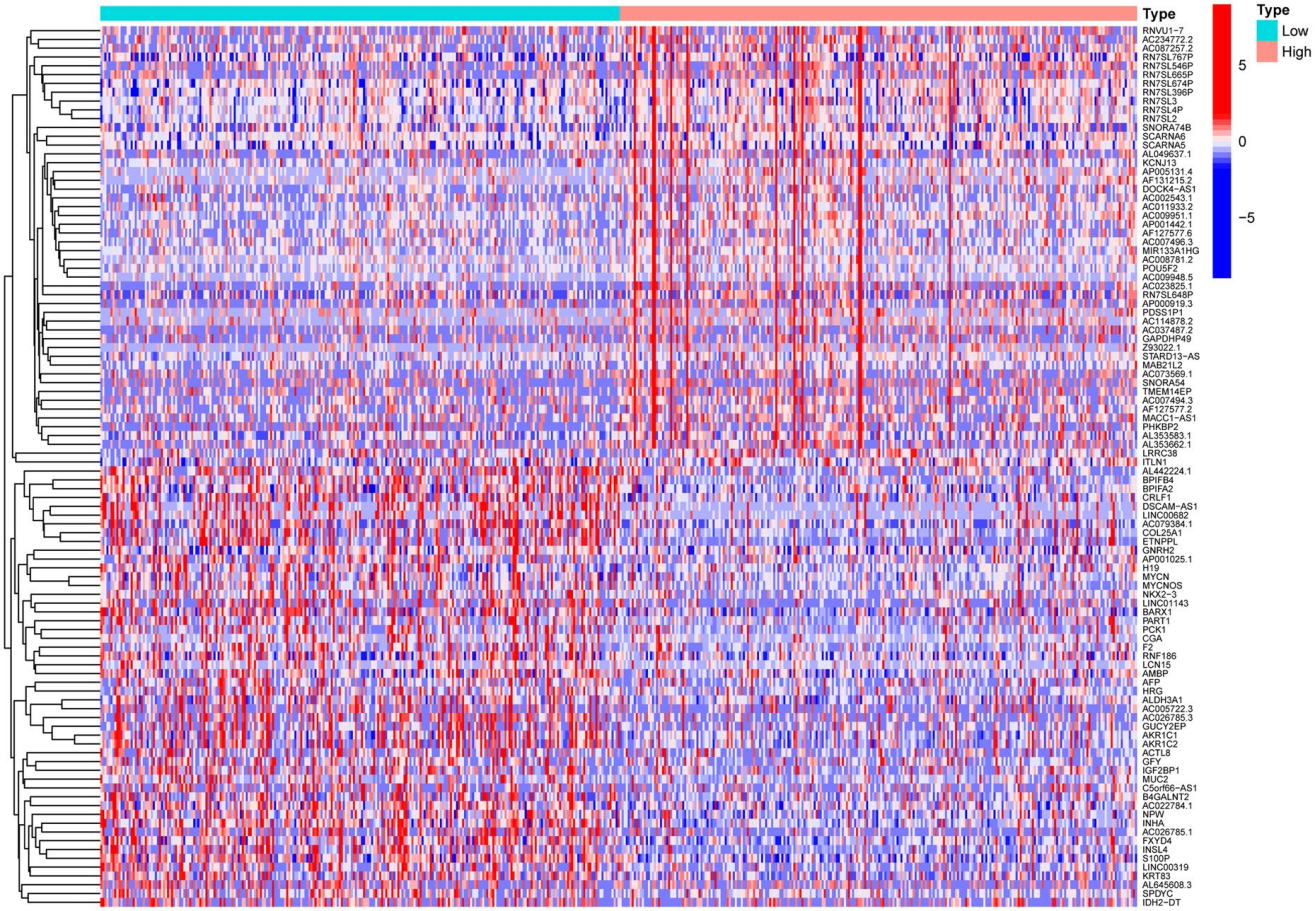
Heatmap showed differentially expressed genes (DEGS) in the CD80 high expression group and CD80 low expression group

Similarly, we divided LUAD patients in TCGA into two groups based on the median CD80 expression. The heatmap (Fig. 8) shows the differentially expressed genes (DEGs) in both groups. A total of 2251 genes were upregulated in the CD80 high expression group, compared to 262 genes upregulated in the low expression group (logFC > 1 or logFC < − 1, *p* < 0.05) (Fig. 8).

**Fig. 9:**
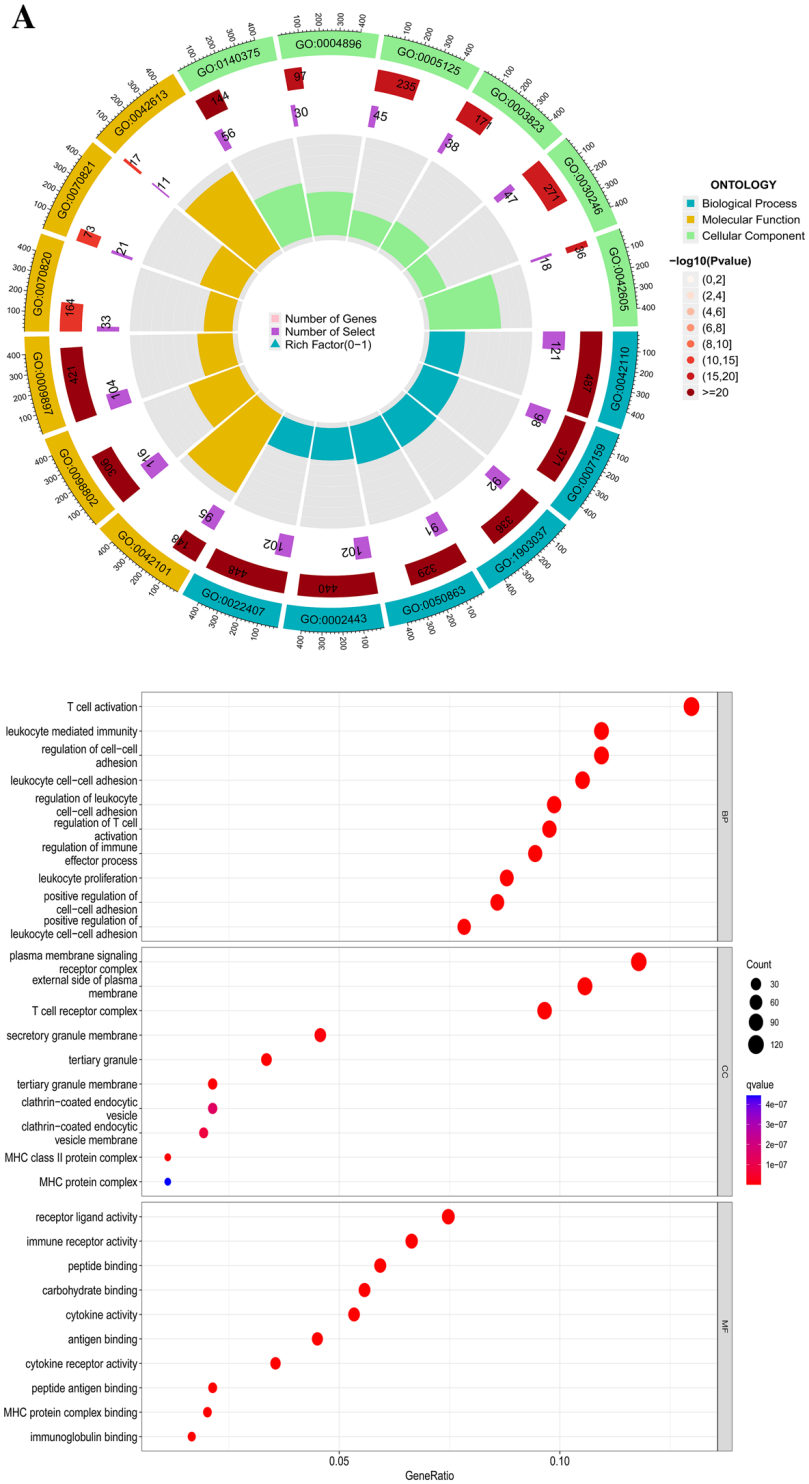

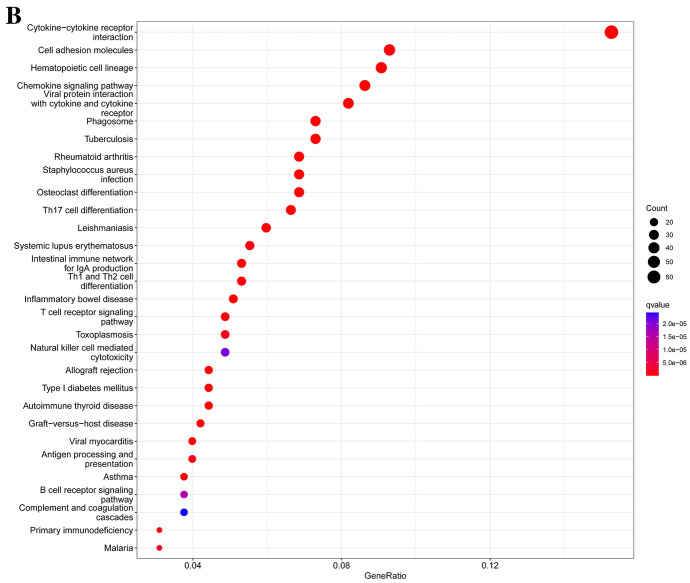
Enrichment analyses of differentially expressed DEGs. **A** GO analysis; **B** KEGG analysis

GO and KEGG analyses were performed to explore the potential functions of differentially expressed genes. Biological process analyses showed that 2513 DEGs were enriched in T-cell activation, leukocyte cell–cell adhesion, regulation of leukocyte cell–cell adhesion, regulation of T-cell activation, leukocyte-mediated immunity, and regulation of cell–cell adhesion. Cellular component analysis showed that the T-cell receptor complex, plasma membrane signaling receptor complex, external side of plasma membrane, tertiary granule, tertiary granule membrane, and MHC class II protein complex were mainly enriched. Molecular function analysis indicated that 2513 DEGs were majorly located in immune receptor activity, cytokine receptor activity, cytokine activity, antigen binding, carbohydrate binding, and peptide antigen binding (Table 1, Fig. 9A). The results of the KEGG pathways analysis indicated that these genes were mainly involved in cytokine–cytokine receptor interaction, cell adhesion molecules, hematopoietic cell lineage, chemokine signaling pathway, viral protein interaction with cytokines and cytokine receptors, and the phagosome (Fig. 9B).

### GSEA

**Fig. 10:**
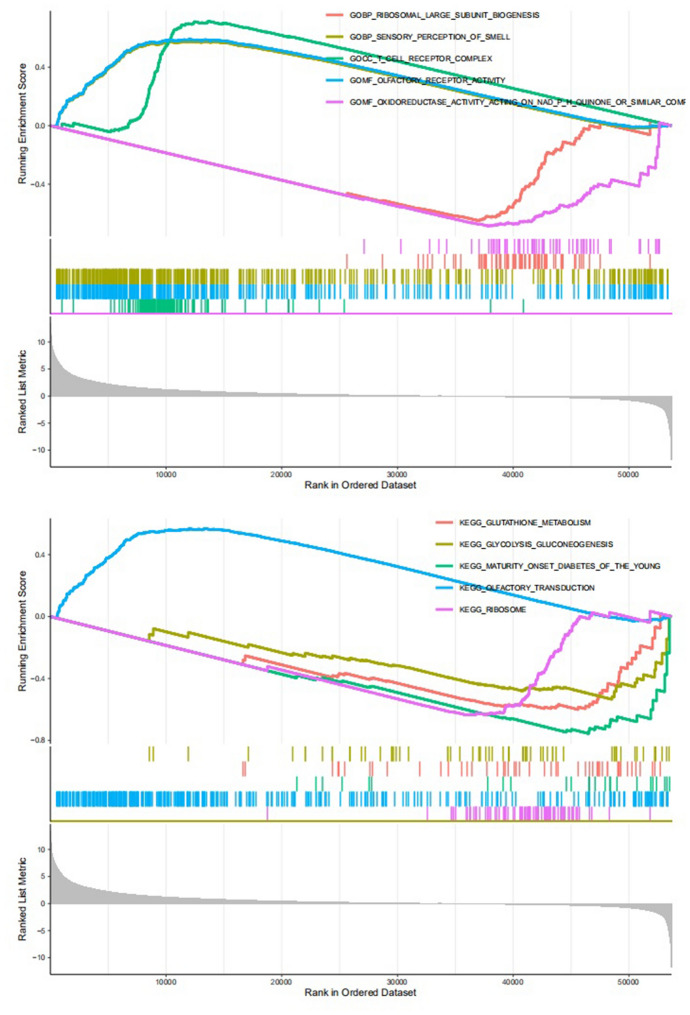
Four significant cell signaling pathways enriched in high CD80 expression by GSEA analysis. Six significant cell signaling pathways enriched in low CD80 expression by GSEA analysis

We performed GSEA analysis and the results showed that sensory perception of smell, T-cell receptor complex, olfactory receptor activity, and olfactory transduction were mainly enriched in the CD80 high expression group. Meanwhile, genes related to ribosomal large subunit biogenesis, oxidoreductase activity acting on NAD(P)H quinone, or similar compound as acceptor, glutathione metabolism, glycolysis, gluconeogenesis, young diabetic onset, and the ribosome were involved in the CD80 low expression group (Fig. 10).

### Tumor Microenvironment

**Fig. 11:**
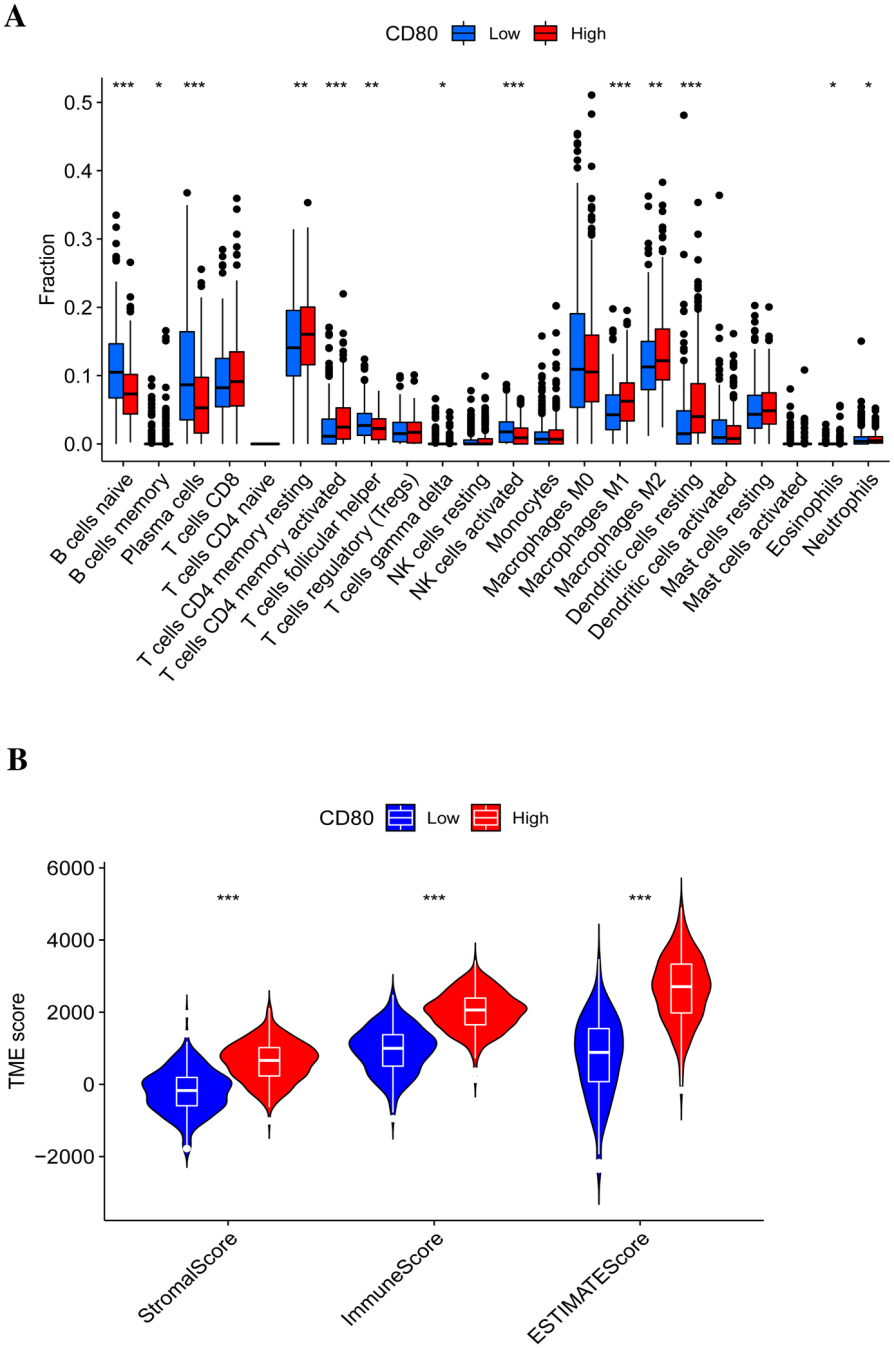
Tumor immune microenvironment analysis: **A** comparison of immune cell infiltration in the CD80 high expression group and low expression group and **B** comparison of immune score and stromal score in the CD80 high expression group and low expression group. *p < 0.05; **p < 0.005; and ***p < 0.001

Any samples obtained from the TCGA database that had a complete gene expression profile and clinical information were able to be incorporated into our study. Using the ESTIMATE algorithm, we calculated that stromal scores were distributed between -1780.4 and 2111.1 and immune scores ranged from -948.8 to 3430.4 (Supplementary file 3 shows the detailed scores for each sample). According to the results, both the immune scores and stromal scores in the CD80 high expression group were much higher than in the CD80 low expression group (Fig. 11A, *p* < 0.05). We therefore hypothesized that CD80 was associated with tumor purity.

**Fig. 12:**
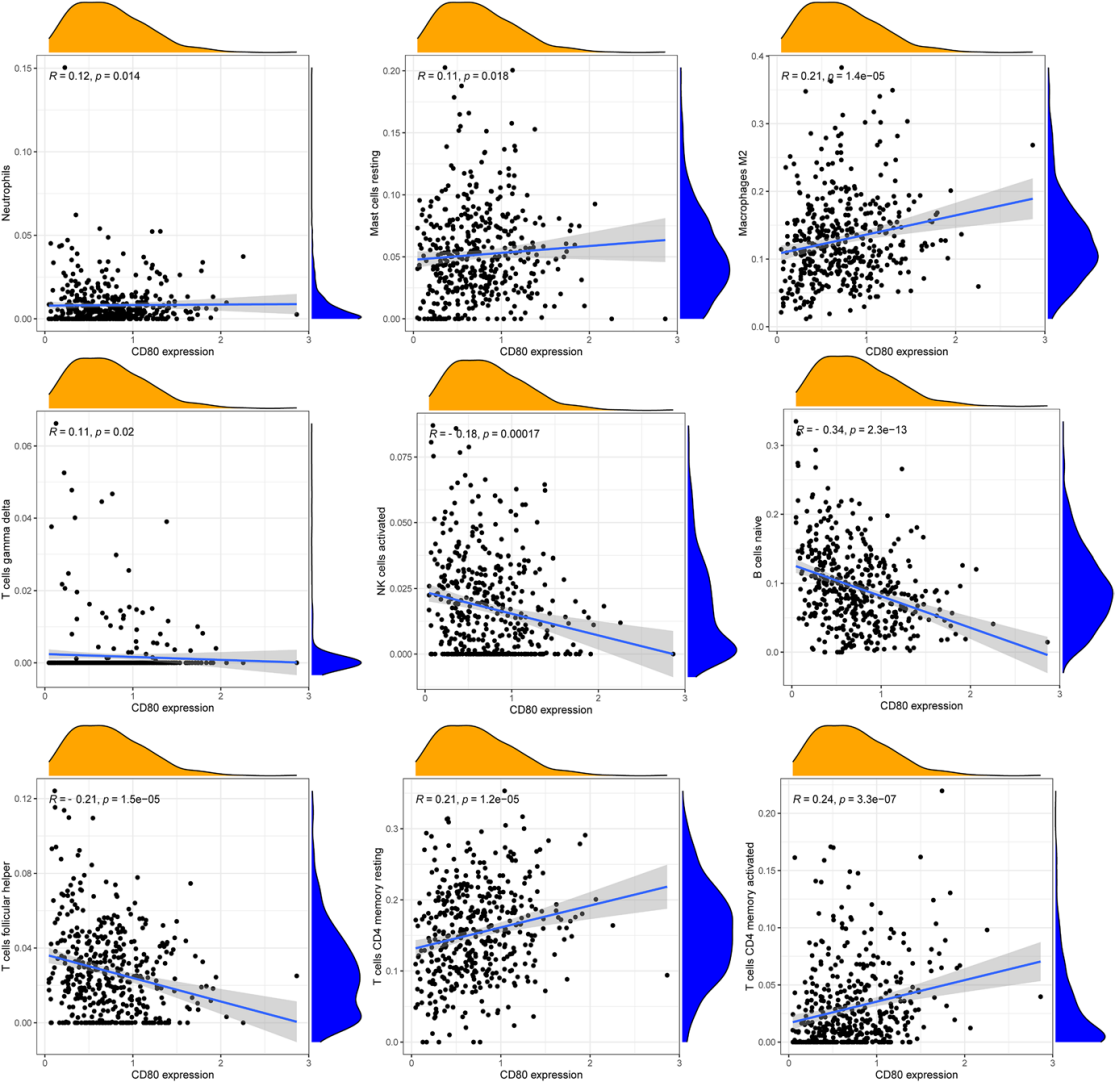

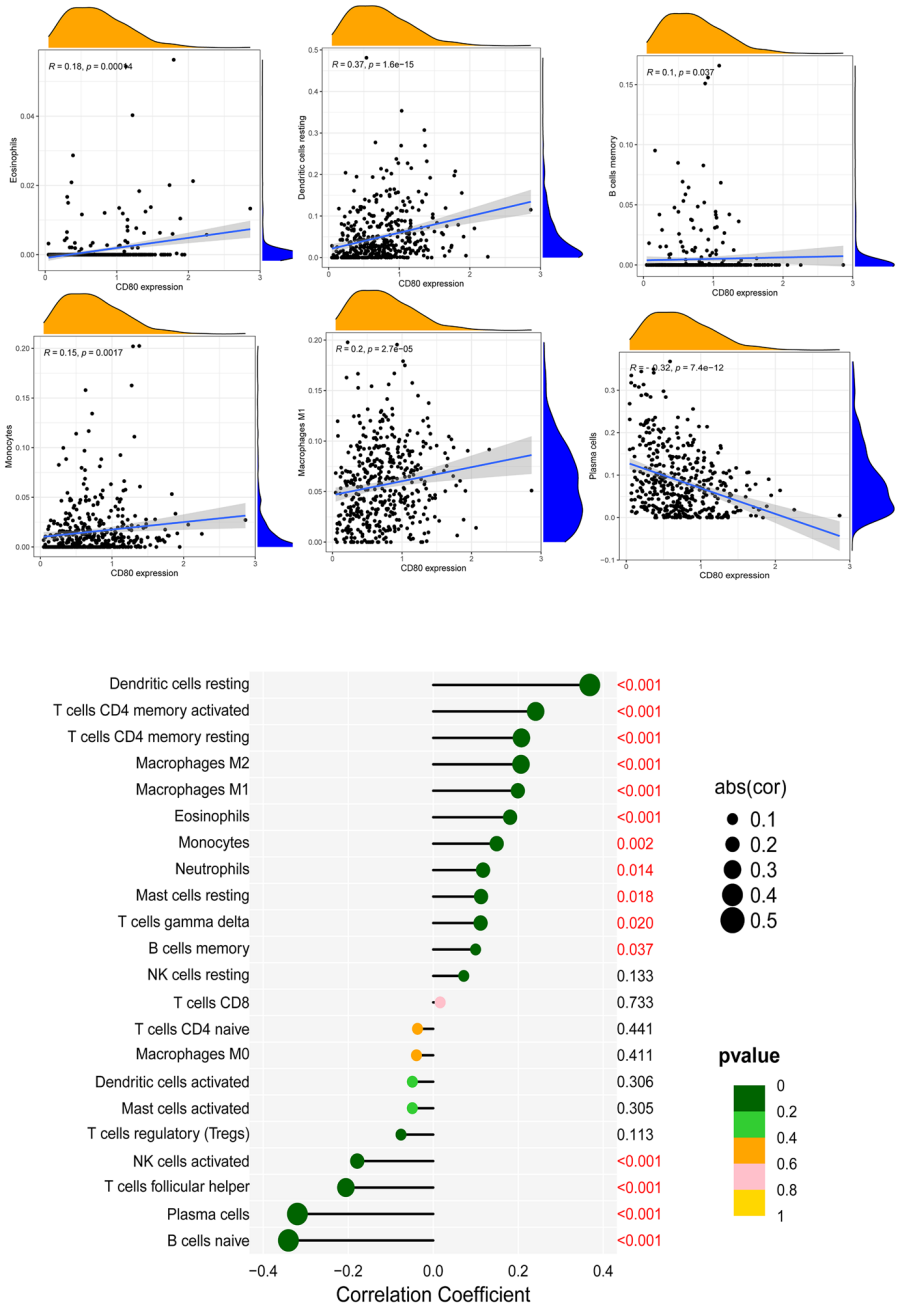
Relevance of different immune cells to CD80 expression in LUAD patients

Next, we used the CIBERSORT algorithm to analyze whether CD80 expression could influence the distribution of infiltrating immune cells in tumor tissues. As the results showed, CD8^+^ T cells, resting memory CD4^+^ T cells, activated memory CD4^+^ T cells, M1 macrophages, M2 macrophages, and resting dendritic cells (DCs) were more enriched in the CD80 high expression LUAD group, while naïve B cells, plasma cells, follicular helper T cells, and activated NK cells were more enriched in the CD80 low expression LUAD group (Fig. 11B, *p* < 0.05). We further investigated the correlation between CD80 expression and immune-infiltrating cells in LUAD (detailed information was available in Supplementary file 2). The results (Fig. 12) showed that CD80 was positively related with memory B cells (*r* = 0.10), γδ T cells (*r* = 0.11), resting mast cells (*r* = 0.11), neutrophils (*r* = 0.12), monocytes (*r* = 0.15), eosinophils (*r* = 0.18), M1 macrophages (*r* = 0.20), M2 macrophages (*r* = 0.21), resting memory CD4^+^ T cells (*r* = 0.21), activated memory CD4^+^ T cells (*r* = 0.24), and resting DCs (*r* = 0.37) and negatively correlated with naïve B cells (*r* = − 0.35), plasma cells (*r* = − 0.32), follicular helper T cells (*r* = − 0.21), and activated NK cells (*r* = − 0.18). All of the above results were statistically significant (*p* < 0.05).

### Association Between CD80 Expression and Immune Checkpoint Inhibitors

**Fig. 13:**
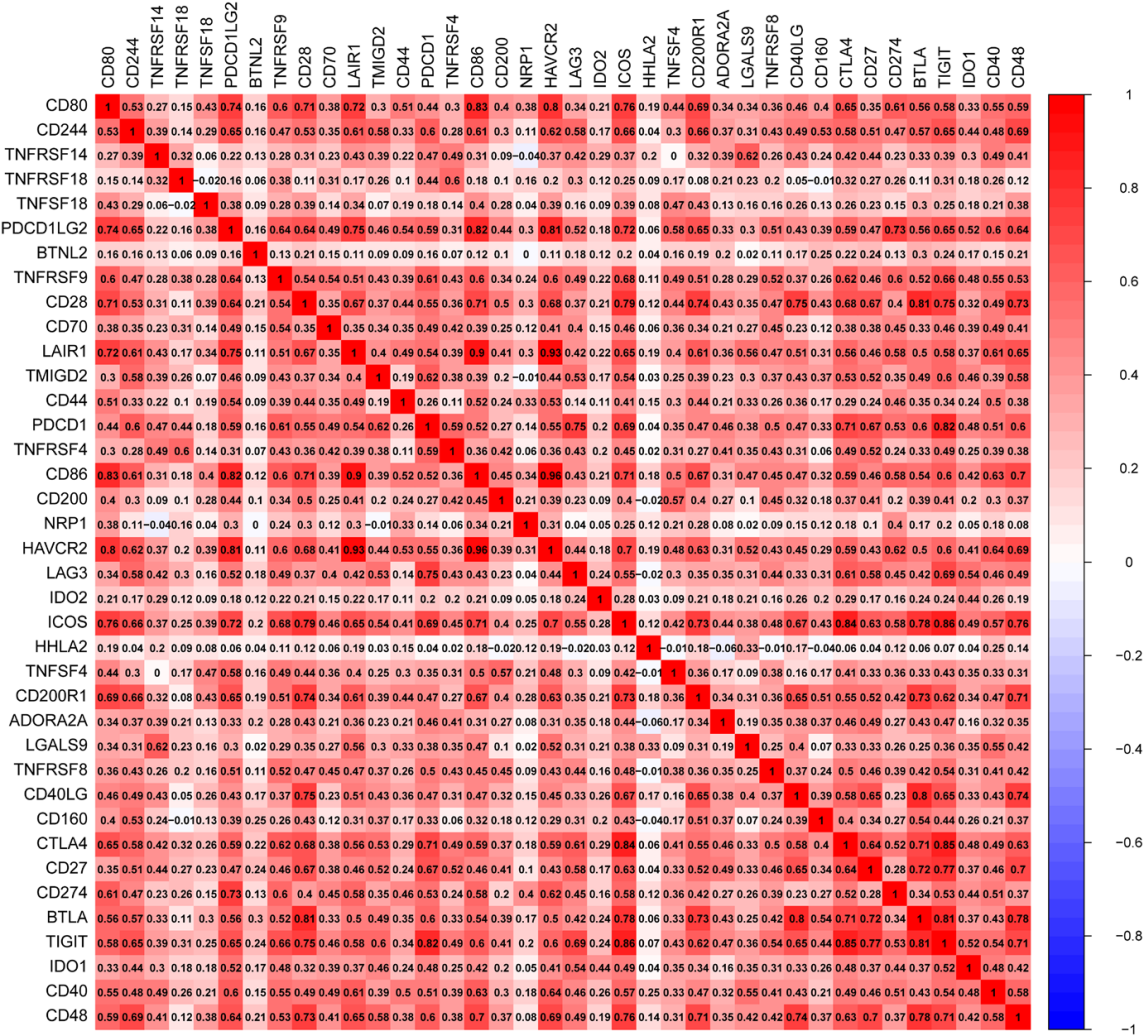
Analysis of the correlation between CD80 and immune checkpoints

Tumor cells achieve immune escape through different pathways for further progression. For example, tumor cells overexpress immunosuppressive checkpoint molecules to impair antitumor immune responses. We already investigated the correlation between CD80 expression and a series of immune checkpoints. The results showed that multiple inhibitory checkpoint molecules, including *HAVCR2*, *PDCD1LG2*, *LAIR1*, *CD200R1*, *CTLA4*, and *CD274*, were positively correlated with CD80 expression levels in LUAD (Fig. 13).

### TMB and Immunotherapy Analysis

**Fig. 14:**
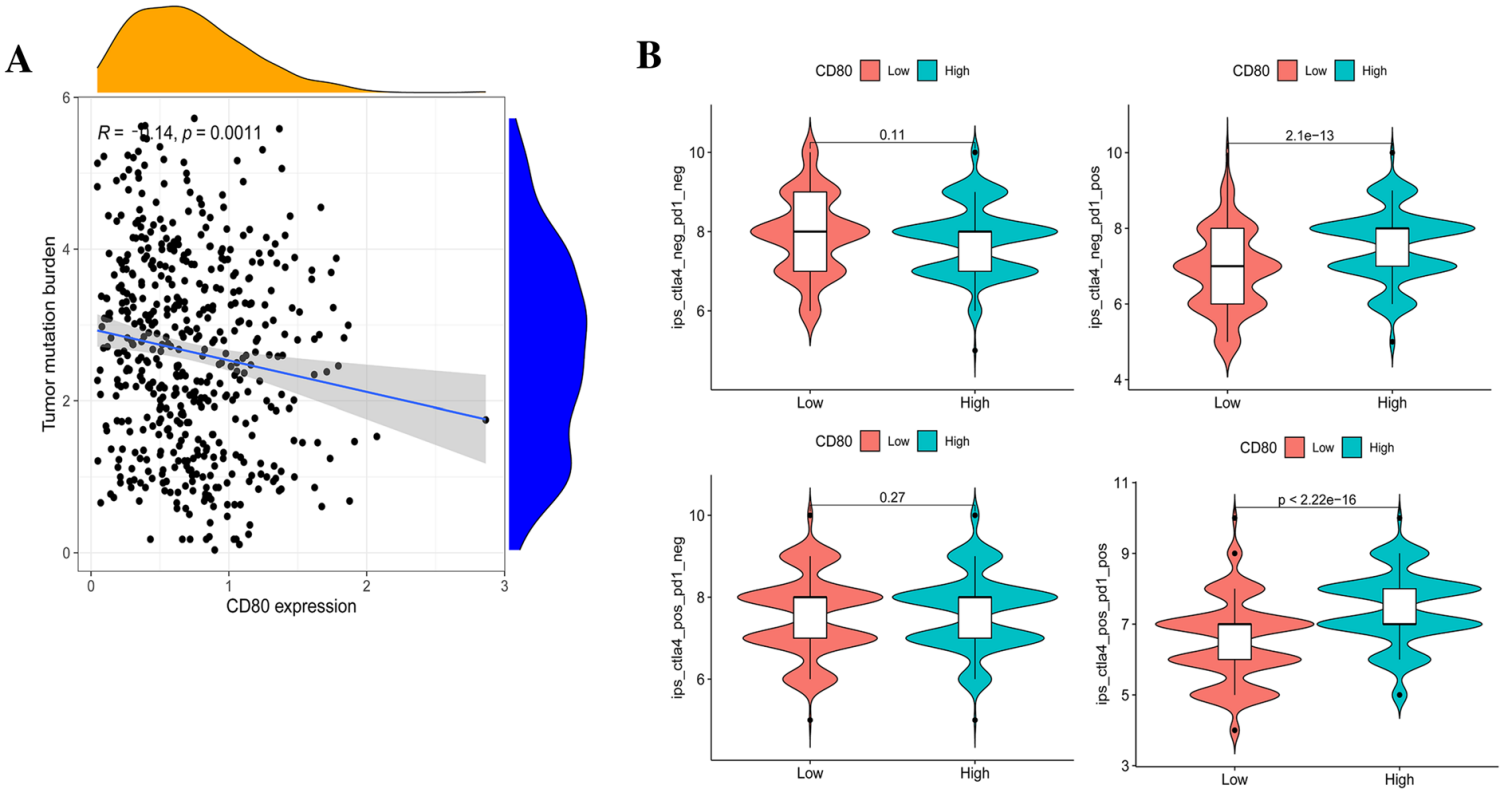
**A** The expression level of CD80 was correlated with the level of TMB in LUAD; **B** differential analysis of immune checkpoint inhibitor sensitivity in the high and low CD80 expression groups

Total mutational burden (TMB) is the number of nonsynonymous mutations in the exon coding regions of the tumor cell genome. It is usually expressed as the total number of substitutions and insertions/deletions of mutations per megabase. Previous literature has shown that a higher tumor mutation burden is associated with the elevated expression of tumor neoantigens on MHC molecules, which help the body’s immune system to recognize foreign substances and generate antitumor immune responses. Although we fortunately found that the TMB level gradually decreased as CD80 expression increased (Fig. 14A), the correlation between CD80 and TMB is low (*r* = − 0.14, *p* = 0.0011). It seems that we need more samples to explore the relationship between CD80 and TMB.

In recent years, ICIs have significantly improved the prognosis of advanced and metastatic LUAD. These agents include antibodies that target CTLA4 or PD-1/PD-L1. To further investigate the sensitivity of CD80 expression to targeted inhibitors and immunotherapy, we obtained the International Prognostic Scores (IPSs) of LUAD cases in TCGA from the TCIA database (https://tcia.at/). The IPS assesses PD-L1 expression on tumor-associated immune cells (lymphocytes, macrophages, etc*.*) and uses PD-L1 expression as a separate evaluation metric to differentiate the beneficiary population. In the case of PD-1/PD-L1 positivity, patients in the CD80 high expression group had a higher IPS (Fig. 14B, *p* < 0.01). This suggests that the cases in these groups (CTLA4^−^PD-1^+^, CTLA4^+^PD-1^+^) may be more sensitive to CTLA4 and/or PD-1/PD-L1 blockade (Tables 1 and 2).

**Table 1:** Ten significant cell signaling pathways enriched in high CD80 expression

ID	NES	*P* value
GOCC_T_CELL_RECEPTOR_COMPLEX	1.740729839	1E−10
GOMF_OLFACTORY_RECEPTOR_ACTIVIT	1.470284116	1.03E−09
GOBP_SENSORY_PERCEPTION_OF_SMEL	1.436432237	5.04E−09
GOBP_RIBOSOMAL_LARGE_SUBUNIT_BI	− 2.433765207	6.31E−09
GOMF_OXIDOREDUCTASE_ACTIVITY_AC	− 2.534860024	1.03E−08
KEGG_RIBOSOME	− 2.393701861	1.16E−10
KEGG_OLFACTORY_TRANSDUCTION	1.401617082	0.000000165
KEGG_MATURITY_ONSET_DIABETES_OF	− 2.199864575	0.0000191
KEGG_GLUTATHIONE_METABOLISM	− 2.089401114	0.0000263
KEGG_GLYCOLYSIS_GLUCONEOGENESIS	− 1.939284954	0.0000462

**Table 2:** Correlation between 10 known genes and CD80 expression (LUAD + normal, *N* = 594)

Gene	Cor	*P* value
MARCH1	0.829052394	1.15E−136
TFEC	0.817515722	7.8161E−130
PTPRC	0.78365837	2.711E−112
TLR4	0.78270612	7.5888E−112
CLEC4A	0.780668708	6.7468E−111
RGS18	0.779668251	1.9559E−110
CD84	0.778253263	8.7293E−110
CYBB	0.778119293	1.0052E−109
GPR65	0.775719656	1.2367E−108
ICOS	0.771844181	6.6743E−107

### Drug Sensitivity Analysis

**Fig. 15:**
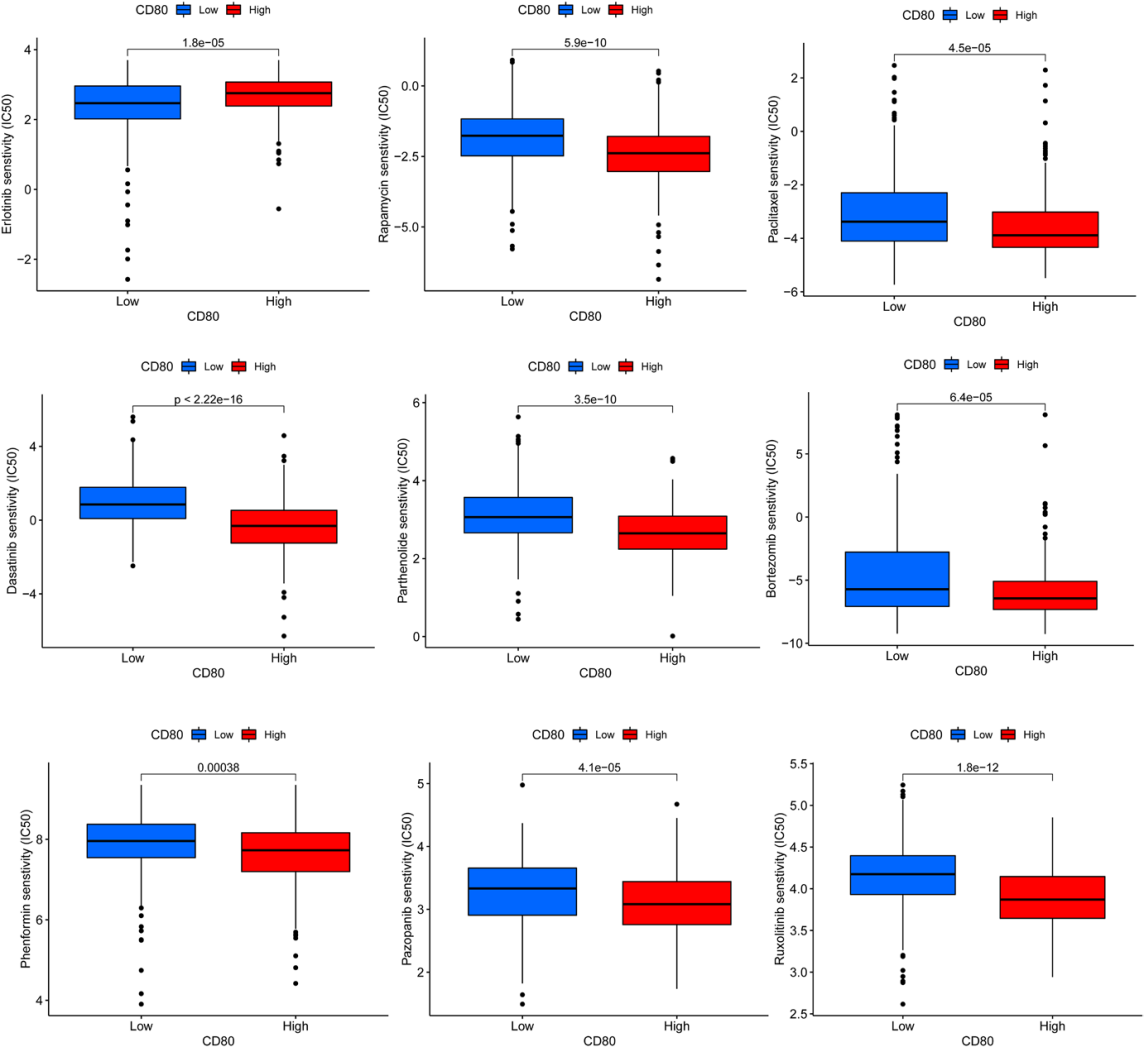

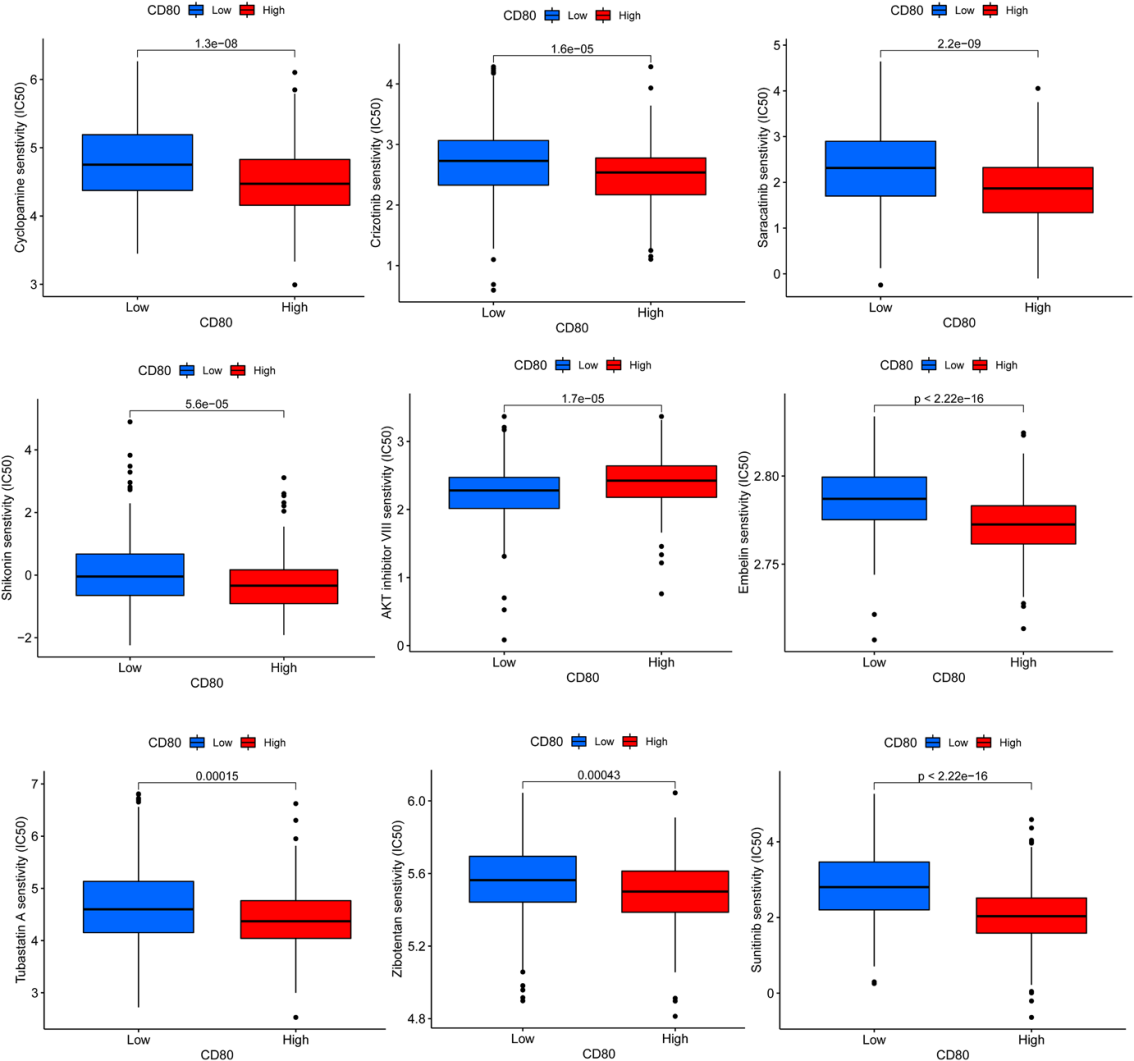
Drug sensitivity analysis

To improve the treatment outcome of LUAD patients, we further investigated the difference in sensitivity to commonly used chemotherapeutic agents and targeted drugs between the two groups. With an R package called pRRophetic, we found that patients in the low expression group had higher IC50 values for rapamycin, sunitinib, paclitaxel, cyclopamine, crizotinib, saracatinib, dasatinib, parthenolide, bortezomib, shikonin, embelin, phenformin, pazopanib, ruxolitinib, tubastatin A, and zibotentan than those in the high expression group. And we suggested that patients in the high CD80 expression group may have been more sensitive to these drugs (Fig. 15). In contrast, the IC50 values of erlotinib and AKT inhibitor VIII were lower in patients of the low expression group (Fig. 15). Therefore, erlotinib and AKT inhibitor VIII may be more effective in treating patients in the low CD80 expression group.

## Discussion

Recently, several biomarkers have been used for the diagnosis and prognosis of malignant tumors, including mRNAs, miRNAs, and proteomic characteristics (Liu et al. 2021; Zhang et al. 2020; Xu et al. 2020). CD80 is a membrane receptor protein that is activated by binding to CD28 or CTLA4. Activation of CD28 induces T-cell proliferation and cytokine production. In recent years, therapies targeting EGFR, ALK, ROS1, MET, and RET, as well as immunotherapy against PD-1/PD-L1, have been used in the treatment of patients with LUAD widely (Ettinger et al. 2019). However, due to the heterogeneity and drug resistance of tumors, new therapeutic approaches are urgently needed.

CD80 has been reported to be correlated with both PD-L1 and PD-L2 in LUAD (Larsen et al. 2019). Related studies have demonstrated that CD80 interacts in a *cis* manner with PD-L1 on antigen-presenting cells (APCs), thereby disrupting PD-L1/PD-1 binding. Thus, when APCs express large amounts of CD80, PD-L1 cannot bind to PD-1 to inhibit T-cell activation (Sugiura et al. 2019). Therefore, it was suggested that CD80 tend to be a potential target for tumor immunotherapy. Interestingly, cells of different tumor types seem to evade antitumor immunity via disparate expression of CD80. In glioblastoma, cancer stem cells lack CD80 expression. This may inhibit co-stimulatory signaling transduced via CD28 expressed on potential antitumor T cells (Huang et al. 2005). In contrast, in cutaneous squamous cell carcinoma, CD80 which is highly expressed by tumor cells has been shown to mediate cancer cell proliferation, T-cell suppression, and failure through contact with CTLA4 (Miao et al. 2019). In spite of this, few studies have provided a comprehensive analysis of CD80 in LUAD.

In our study, we first explored the prognostic value of CD80 expression in LUAD. The expression of CD80 was significantly lower in LUAD than in the normal group, indirectly suggesting that CD80 may inhibit tumorigenesis. This was further validated in our research, as we found that CD80 overexpression significantly improved OS in LUAD patients. Next, we further investigated the relationship between CD80 expression and individual clinical features. Multivariate COX analysis confirmed that reduced CD80 expression was an important independent risk factor for the development of LUAD and was associated with age and sex, but not with tumor stage. To further predict the survival of LUAD patients, we constructed a nomogram consisting of CD80 expression and clinical features to predict the 1-, 3-, and 5-year survival rates of LUAD patients. This was verified by calibration curves, which showed that the actual survival of patients was consistent with the predicted values.

A growing number of cell signaling pathways have been shown to participate in tumorigenesis and progression. These signaling pathways could be used as targets for biological antitumor therapy (Clara et al. 2020). We found that high CD80 expression was associated with multiple cellular signaling pathways by GSEA analysis, including T-cell receptor (TCR) signaling. The TCR receives foreign antigens provided by antigen-presenting cells as an important part of the body’s immune response (Alcover et al. 2018), and CD80 may participate in regulating the TCR complex. In contrast, tumor samples with low expression of CD80 were enriched in genes associated with ribosomal large subunit biogenesis, oxidoreductase activity acting on NAD(P)H quinone or similar compound as an acceptor, glutathione metabolism, glycolysis, and gluconeogenesis. Ribosomes are composed of many different proteins and nucleic acids involved in protein synthesis. Dysfunction or changes in the number of ribosomes can lead to deviations in protein translation patterns, which may ultimately promote tumorigenesis or progression (Pelletier et al. 2018). Tumor cells are able to adapt to higher levels of reactive oxygen species (ROS) either by activating antioxidant transcription factors or by increasing NADPH through various pathways, such as the pentose phosphate pathway (PPP) or glutamine metabolism (Hayes et al. 2020). Tumor cells also participate in gluconeogenic and glycolytic pathways by utilizing or regulating glycoisomeric enzymes to grow and survive in a specific metabolic microenvironment (Grasmann et al. 2019). CD80 may be involved in these signaling pathways and contribute to the malignant progression of LUAD.

The activation of oncogenes and inactivation of tumor suppressor genes are particularly important in cancer development (Kontomanolis et al. 2020). By gene co-expression analysis, we found 10 genes most associated with CD80 expression, including five positively and five negatively associated genes. Among the top three negative associations were *GSTP1*, *PELP1*, and *ECH1*. These three genes are particularly important in the development of various diseases. In prostate cancer, epigenetic silencing of the glutathione-S-transferase P1 (GSTP1) gene may be important in the development of tumors (Henrique and Jerónimo 2004). Changes in the localization of proline-, glutamate-, and leucine-rich protein 1 (PELP1) in the cytoplasm are an oncogenic event that promote breast cancer initiation and progression (Truong et al. 2018). Downregulation of enoyl-CoA hydratase 1 (ECH1) inhibits Hca-F cells’ ability to metastasize to peripheral lymph nodes in vivo, thereby inhibiting the development and progression of tumor metastasis (Zhang et al. 2013). Among the top five positive associations were *MARCH1*, *TLR4*, and *CLEC4A*. In hepatocellular carcinoma, MARCH1 regulates the PI3K-AKT-β-catenin pathway, thereby promoting tumor progression (Xie et al. 2019). Epithelial toll-like receptor 4 (TLR4) signaling activates dual oxidase 2 (DUOX2) to induce microbiota-driven colitis-associated tumorigenesis (Burgueño et al. 2021). C-type lectin domain family 4, member A (CLEC4A) is an immunosuppressive factor for DCs. Thus, CD80 was both positively and negatively associated with various pro-tumorigenic factors, which may offer insight into potential therapeutic targets in both the CD80 high and low LUAD subsets.

To improve the therapeutic efficacy of ICIs and to explore new biological antitumor treatment strategies, we studied the distribution of infiltrating immune cells in LUAD tissues. In our study, CD8^+^ T cells, resting memory CD4^+^ T cells, activated memory CD4^+^ T cells, M1 macrophages, and M2 macrophages were more abundant in the CD80 high subpopulation, whereas follicular helper T cells were more abundant in the CD80 low subgroup. Tumor-specific CD4^+^ and CD8^+^ effector T cells effectively kill cancer cells and are critical for an effective antitumor response. CD4^+^ T cells recruit tumor-specific CD8^+^ T cells and activate CD8^+^ effector T cells and NK cells to kill tumor cells. Previous lectures have reported that intensive infiltration by CD8^+^ T cells and M1 macrophages indicates good prognosis (Gentles et al. 2015; Fridman et al. 2017; Garrido-Martin et al. 2020). In addition, in gastric, breast, bladder, prostate, and lung cancers, M2 macrophages have been reported to be related to the development of tumor growth and aggressive phenotypes, ultimately leading to poor prognosis in gastric, breast, bladder, prostate, and lung cancers (Ruffell and Coussens 2015; Pathria et al. 2019).

Tumor immune escape allows tumor cells to avoid clearance by the body and use nonspecific inflammation to spread through multiple mechanisms, leading to proliferation and metastasis. This process has been highly regarded in cancer research in recent years, especially in relation to the role of PD-1 and PD-L1 (Jiang et al. 2019). As our results showed, CD80 expression was also positively correlated with immune checkpoints, such as CD274 and CTLA4. Therefore, patients with high CD80 expression may likely benefit from immune checkpoint inhibitors, such as antibodies targeting CTLA4 or PD-1/PD-L1. Using the IPSs of LUAD patients in the TCIA database, we further validated the conjecture that patients co-expressing CD80 and PD-1 have better outcomes for immunotherapy.

Through the pRRophetic analysis, we found that LUAD patients with high CD80 expression may benefit from treatment with rapamycin, paclitaxel, crizotinib, and bortezomib, while patients with LUAD with low CD80 expression may benefit from treatment with erlotinib and AKT inhibitors. By altering molecular pathways, some small-molecule drugs are able to enhance immune response while eliminating immunosuppression and tolerance. Thus, small-molecule drugs can synergize with ICIs to enhance their efficacy (Han et al. 2021; Zanden et al. 2020). Rapamycin, an inhibitor of mechanistic target of rapamycin complex (mTORC), blocks mTOR functions and yields antiproliferative activity in a variety of malignancies (Vignot et al. 2005). Paclitaxel promotes the assembly of microtubule proteins into microtubules, prevents microtubule binding to microtubules, prevents microtubule dissociation, blocks cell cycle progression, prevents mitosis, inhibits the growth of cancer cells, directly kills tumor cells, and modulates various immune cells (Zhu and Chen 2019). Crizotinib, an ATP-competitive small-molecule inhibitor of receptor tyrosine kinases C-Met, ALK, and ROS1, has shown significant efficacy in patients with advanced ALK-positive lung cancer (Shaw et al. 2020). Bortezomib’s ability to favorably modulate apoptosis-related protein expression and its moderate toxicity as a single agent provide the basis for its combination with cytotoxic agents in the treatment of lung cancer (Davies et al. 2007). The EGFR inhibitor erlotinib reduced CD4^+^ effector regulatory T-cell infiltration in the tumor microenvironment and showed better antitumor effects in combination with αPD-1 monoclonal antibody than either treatment alone (Sugiyama et al. 2020). AKT inhibitors result in superior antitumor effects in minor histocompatibility antigen-specific T cells (Waart et al. 2014). Thus, based on our data, the use of these small molecular drugs in combination with immune checkpoint blockade to enhance and improve the efficacy of LUAD bears further investigation.

There are still shortcomings in our study. First, the samples in the TCGA database were mainly tumor samples, with only a small number of normal tissues, which may produce bias. Second, although CD80 benefited LUAD patients from immunotherapy, the expression of CD80 was negatively correlated with TMB. This is likely because patients with high CD80 expression efficiently clear mutated cells; thus any cancer that survives in such individuals must have a low TMB or else be eliminated by the immune system. However, the correlation between CD80 and TMB is low, and we need more samples to make sure whether CD80 was negatively correlated with TMB. Such a hypothesis, of course, will need to be further verified by relevant experiments. In conclusion, although we have explored the role of CD80 in LUAD, more systematic experiments are needed to verify our conclusions.

## Conclusion

Despite the involvement of the CD80-encoded protein in tumorigenesis and progression in conjunction with CTLA4, we still found that CD80 improves the prognosis of LUAD patients. Patients with high CD80 expression may benefit from immune checkpoint inhibitors, such as antibodies targeting CTLA4 or PD-1/PD-L1. These findings suggest that CD80 is likely to be a potential target for improving the prognosis of LUAD patients and the efficacy of biological antitumor therapy. In the meantime, small molecular drugs discovered through our study may improve the treatment of LUAD in future.

## Supplementary Information

Supplementary file 1 shows the results of immune cell infiltration calculated by CIBERSORT for each sample. The detailed information between CD80 expression and immune-infiltrating cells in LUAD are available in Supplementary file 2. Supplementary file 3 shows the detailed stromal scores, immune scores, and estimate scores for each sample. Supplementary file 4 describes the CD80 expression of each sample.

### Supplementary Information

Below is the link to the electronic supplementary material.

Supplementary file1 (TXT 210 KB)

Supplementary file2 (TXT 1 KB)

Supplementary file3 (TXT 52 KB)

Supplementary file4 (TXT 31 KB)

## Data Availability

Transcriptome data can be obtained from the TCGA database (https://portal.gdc.cancer.gov/), and raw data for further analysis can be obtained from the email address of the contributing author, Feng Wei (E-mail: 20205232148@stu.suda.edu.cn).

## References

[CR1] Alcover A, Alarcón B, Di Bartolo V (2018). Cell biology of T cell receptor expression and regulation. Annu Rev Immunol.

[CR2] Burgueño JF (2021). Epithelial TLR4 signaling activates DUOX2 to induce microbiota-driven tumorigenesis. Gastroenterology.

[CR3] Butte MJ (2007). Programmed death-1 ligand 1 interacts specifically with the B7–1 costimulatory molecule to inhibit T cell responses. Immunity.

[CR4] Chaudhri A (2018). PD-L1 binds to B7–1 only on the same cell surface. Cancer Immunol Res.

[CR5] Chen J (2020). Genomic landscape of lung adenocarcinoma in East Asians. Nat Genet.

[CR6] Clara JA (2020). Targeting signalling pathways and the immune microenvironment of cancer stem cells—a clinical update. Nat Rev Clin Oncol.

[CR7] Davies AM (2007). Incorporating bortezomib into the treatment of lung cancer. Clin Cancer Res.

[CR8] Ettinger DS (2019). NCCN guidelines insights: non-small cell lung cancer, version 1.2020. J Natl Compr Cancer Netw: JNCCN.

[CR9] Fridman WH (2017). The immune contexture in cancer prognosis and treatment. Nat Rev Clin Oncol.

[CR10] Garrido-Martin EM (2020). M1 tumor-associated macrophages boost tissue-resident memory T cells infiltration and survival in human lung cancer. J Immunother Cancer.

[CR11] Gentles AJ (2015). The prognostic landscape of genes and infiltrating immune cells across human cancers. Nat Med.

[CR12] Grasmann G (2019). Gluconeogenesis in cancer cells—repurposing of a starvation-induced metabolic pathway?. Biochim Biophys Acta.

[CR13] Han C (2021). Small molecular drugs reshape tumor microenvironment to synergize with immunotherapy. Oncogene.

[CR14] Hayes JD, Dinkova-Kostova AT, Tew KD (2020). Oxidative stress in cancer. Cancer Cell.

[CR15] Henrique R, Jerónimo C (2004). Molecular detection of prostate cancer: a role for GSTP1 hypermethylation. Eur Urol.

[CR16] Horn L (2018). First-line atezolizumab plus chemotherapy in extensive-stage small-cell lung cancer. N Engl J Med.

[CR17] Huang J (2005). The Hippo signaling pathway coordinately regulates cell proliferation and apoptosis by inactivating Yorkie, the Drosophila Homolog of YAP. Cell.

[CR18] Jiang X (2019). Role of the tumor microenvironment in PD-L1/PD-1-mediated tumor immune escape. Mol Cancer.

[CR19] Kontomanolis EN (2020). Role of oncogenes and tumor-suppressor genes in carcinogenesis: a review. Anticancer Res.

[CR20] Larsen TV, Hussmann D, Nielsen AL (2019). PD-L1 and PD-L2 expression correlated genes in non-small-cell lung cancer. Cancer Commun (london, England).

[CR21] Li H (2020). Efficacy of cascade-primed cell infusion as an adjuvant immunotherapy with concurrent chemotherapy for patients with non-small-cell lung cancer: a retrospective observational study with a 5-year follow-up. Cytotherapy.

[CR22] Liu X-S (2021). NPM1 is a prognostic biomarker involved in immune infiltration of lung adenocarcinoma and associated with m6A modification and glycolysis. Front Immunol.

[CR23] Miao Y (2019). Adaptive immune resistance emerges from tumor-initiating stem cells. Cell.

[CR24] Pathria P, Louis TL, Varner JA (2019). Targeting tumor-associated macrophages in cancer. Trends Immunol.

[CR25] Paz-Ares L (2019). Durvalumab plus platinum-etoposide versus platinum-etoposide in first-line treatment of extensive-stage small-cell lung cancer (CASPIAN): a randomised, controlled, open-label, phase 3 trial. Lancet (london, England).

[CR26] Pelletier J, Thomas G, Volarević S (2018). Ribosome biogenesis in cancer: new players and therapeutic avenues. Nat Rev Cancer.

[CR27] Quintanal-Villalonga Á (2020). Lineage plasticity in cancer: a shared pathway of therapeutic resistance. Nat Rev Clin Oncol.

[CR28] Ruffell B, Coussens LM (2015). Macrophages and therapeutic resistance in cancer. Cancer Cell.

[CR29] Shaw AT (2020). First-line lorlatinib or crizotinib in advanced -positive lung cancer. N Engl J Med.

[CR30] Sugiura D (2019). Restriction of PD-1 function by -PD-L1/CD80 interactions is required for optimal T cell responses. Science (new York, N.y.).

[CR31] Sugiyama E (2020). Blockade of EGFR improves responsiveness to PD-1 blockade in -mutated non-small cell lung cancer. Sci Immunol.

[CR32] Truong TH (2018). Cancer stem cell phenotypes in ER breast cancer models are promoted by PELP1/AIB1 complexes. Mol Cancer Res: MCR.

[CR33] van der Waart AB (2014). Inhibition of Akt signaling promotes the generation of superior tumor-reactive T cells for adoptive immunotherapy. Blood.

[CR34] van der Zanden SY (2020). Opportunities for small molecules in cancer immunotherapy. Trends Immunol.

[CR35] Vignot S (2005). mTOR-targeted therapy of cancer with rapamycin derivatives. Ann Oncol.

[CR36] Xie L (2019). MARCH1 encourages tumour progression of hepatocellular carcinoma via regulation of PI3K-AKT-β-catenin pathways. J Cell Mol Med.

[CR37] Xu J-Y (2020). Integrative proteomic characterization of human lung adenocarcinoma. Cell.

[CR38] Yang C-Y, Yang JC-H, Yang P-C (2020). Precision management of advanced non-small cell lung cancer. Annu Rev Med.

[CR39] Yang W-C, Hsu F-M, Yang P-C (2020). Precision radiotherapy for non-small cell lung cancer. J Biomed Sci.

[CR40] Zhang J (2013). Ech1 is a potent suppressor of lymphatic metastasis in hepatocarcinoma. Biomed Pharmacother Biomed Pharmacother.

[CR41] Zhang C (2019). Emerging therapies for non-small cell lung cancer. J Hematol Oncol.

[CR42] Zhang Y (2020). Expression and prognostic significance of m6A-related genes in lung adenocarcinoma. Med Sci Monit.

[CR43] Zhou Z-R (2019). In-depth mining of clinical data: the construction of clinical prediction model with R. Ann Transl Med.

[CR44] Zhu L, Chen L (2019). Progress in research on paclitaxel and tumor immunotherapy. Cell Mol Biol Lett.

